# Neurosurgical Patients’ Experiences and Surgical Outcomes Among Single Tertiary Hospitals in Ethiopia and the United States

**DOI:** 10.7759/cureus.22035

**Published:** 2022-02-08

**Authors:** Justus Boever, Trisha Weber, Eric A Krause, Jemal A Mussa, Yetsedaw G Demissie, Abraham T Gebremdihen, Fassil B Mesfin

**Affiliations:** 1 Neurological Surgery, University of Missouri School of Medicine, Columbia, USA; 2 Neurological Surgery, Aabet Hospital, Addis Ababa, ETH

**Keywords:** intensive care unit, low- and middle-income country, anterior cervical discectomy fusion, sub-saharan africa, estimated blood loss, length of hospital stay (los)

## Abstract

Background

In 2020, we published findings on reported outcomes of anterior cervical decompression and fusion surgery among neurosurgeons in Africa and North America. We found more similarities in outcomes than expected, however, differences still existed. Most notable was the length of stay of patients postoperatively in Africa compared to North America. We sought to examine the neurosurgical practices more closely at a single hospital in Ethiopia and compare it to our own institution, the University of Missouri in Columbia (UMC).

Methods

Two authors spent one week at Aabet Hospital (AH) in Ethiopia. Throughout the week, one author rotated in the clinic and OR gathering the information. Data collection for patients at UMC was collected through retrospective chart review over one week.

Results

A total of eight elective surgeries and four emergency procedures occurred at AH and 18 clinic patients were included in the study. The intraoperative data was collected during the elective procedures at AH. At UMC there were 99 clinic patients, and 29 elective surgeries and one emergency procedure were performed. Procedures at both institutions included cranial, spinal, vascular, and implantable/other cases. Distance travelled by patients to UMC was an average of 57 miles compared to 85 miles at AH. The median pre-op and post-op stays at AH were 2.5 and 6 days compared to 0.2 and 2.1 at UMC, respectively. Blood loss was greater at AH with a median blood loss of 175 mL. Median blood loss at UMC was 50 mL.

Conclusion

We found notable differences among neurosurgical practice and patient demographics at AH compared to UMC. This information will serve as the cornerstone for gathering more information about neurosurgical practice in Ethiopia where electronic medical records are unavailable.

## Introduction

In 2006, the Neurosurgery Education and Development (NED) Foundation (NEDF) began holding surgical camps throughout East Africa [1]. This same year, a Norwegian neurosurgical group started developing a neurosurgical training program in Ethiopia. In 2010, there were only two neurosurgeons in Ethiopia [2]. Now, there are over 25, with several others currently being trained. The need for such training programs in Ethiopia, and, indeed, all Sub-Saharan African (SSA) countries, cannot be overstated. It is estimated that there is one neurosurgeon per roughly 7 million people in SSA [3]. Additionally, in a population of approximately 356 million people, there is an estimated unmet surgical need of just under 5000 people per 100,000 [4]. In 2010, 32.9% of all global deaths were due to conditions requiring surgical intervention. This outpaced the combined yearly deaths of HIV/AIDS, malaria, and tuberculosis by over 4 to 1 [5,6]. It has also been noted that the establishment of emergent and essential surgery in Africa rivals childhood vaccination in terms of economic benefit [7]. Furthermore, roughly 10% of all deaths worldwide are due to trauma, the vast majority of which occur in low and middle-income countries (LMIC), and of which traumatic brain injury and spinal cord injury serve as the most common cause of death and disability [8,9]. This problem is compounded by the fact that in LMIC, increased use of motorized vehicles is not equally met with improved roads and adequate infrastructure [10,11].

Given the relative newness of neurosurgery in SSA and the importance of its advancement, we have sought to examine its progress. In 2020, we published findings on the reported outcomes of anterior cervical decompression and fusion (ACDF) surgery among neurosurgeons in Africa and North America. The results demonstrated more similarities in outcomes than was expected, however, some differences still existed. The starkest difference was on the length of stay (LOS) of patients postoperatively in Africa compared to North America. Of the 44 African-practicing respondents, 40 reported that the average LOS was a minimum of two days, compared to 14 of 15 North American respondents who reported an average stay of one day or less [12].

Since the findings of that study, we wanted to examine the neurosurgical practices more closely at a single hospital in Ethiopia and then compare it to our own institution, the University of Missouri in Columbia (UMC). By evaluating and comparing perioperative patient information, we hoped to further elucidate differences among the two neurosurgical departments, why such differences exist. This should aid in developing innovations where required. Because of the lack of electronic medical records throughout much of Africa, our travel was necessary to collect data in Ethiopia. Furthermore, the authors at UMC wished to avoid placing any additional burden or tasks on residents. Lastly, travel allowed for in-person collaboration and partnership development. This study serves as an initiative to collect patient and operative data at a single hospital in Addis Ababa, Ethiopia which is highly necessary as the neurosurgeons there, and most hospitals throughout SSA, do not have any means of tracking such information without laboriously copying paper charts into their own personal spreadsheets.

This article was previously presented as a poster at the 2021 Health Science Research Day on November 19, 2021.

## Materials and methods

Two authors (Boever, Mesfin) spent the week of May 17-21, 2021 at Aabet Hospital (AH) in Addis Ababa, Ethiopia. Lack of electronic medical records in Ethiopia made retrospective chart review impossible and, therefore, prospective data gathering was done at AH. Throughout the week, one author (Boever) spent time rotating in the clinic and operating room. The neurosurgical department holds elective surgeries every weekday, usually consisting of two elective procedures per day, though more complex cases may lead to the cancellation of the second surgery. Clinic days are on Monday and Friday. The clinic itself is a single room with three desks and three computers where four residents are constantly seeing patients. Charting is done exclusively on paper and there is no patient database. There is one exam table in the room. Answers were recorded from postoperative follow-up patients including: sex, age, distance traveled (in km), pre-operative length of stay in hospital, post-operative length of stay in hospital, and reason for the operation.

The same author also collected information in the operating room. This information included the time out and time of closing, age, sex, operation, and estimated blood loss (EBL). In Ethiopia, due to surgeries and clinic patients being attended to simultaneously at times, a small number of patients (less than five) were unable to have information collected regarding their surgery or perioperative experience.

Data collection for patients at UMC was done through a retrospective chart review. The week of April 12-16, 2021, was chosen as the best week of comparison due to a typical clinic volume and all neurosurgeons operating on a normal schedule. All patients seen by a neurosurgeon in either department were included. Any surgeries done by orthopedic spine surgeons, however, were excluded. All information collected was placed in a Microsoft Excel (Microsoft® Corp., Redmond, WA) data sheet and then analyzed using Excel data analysis. Analyses included range, median, and mean values (where applicable).

## Results

A total of 12 surgeries occurred at AH and 18 patients seen in the clinic were included in the study. Of the 12 surgeries at AH, four were emergency procedures that occurred at night while the residents were on call. The other eight surgeries were all elective cases that occurred between Monday and Friday. It was the elective cases that allowed for intraoperative data to be collected. In the comparison week at UMC, there were 99 patients seen in the clinic and 30 surgeries performed. Surgeries and patient post-op clinics were categorized into the following: spinal, cranial, vascular, implantable device/other with the results shown in Table 1. AH has eight neurosurgical residents and four attending surgeons. UMC has seven residents and five attending surgeons. One author (Mesfin), a neurosurgical spine surgeon, assisted in several surgeries while at AH during the week.

**Table 1:** Categorization of surgeries performed and post-op clinic visits at University of Missouri in Columbia (UMC) and Aabet Hospital (AH). * = One cranial case at UMC was an emergent procedure and information from that case did not contribute to our analyses. ** = All cranial cases done during the week of data collection were emergency procedures done at night (after the authors had left the hospital) and thus no intraoperative data from those procedures was collected. The indications for those four surgeries were (1) brain abscess, (1) epidural hematoma, and (2) depressed skull fracture.

	Operations			Clinic	
	UMC	AH		UMC	AH
Spine	14	7		44	5
Cranial	8*	4**		37	11
Vascular	4	0		9	1
Implantable devices/Other	4	1		9	1
Total	30	12		99	18

**Figure 1:**
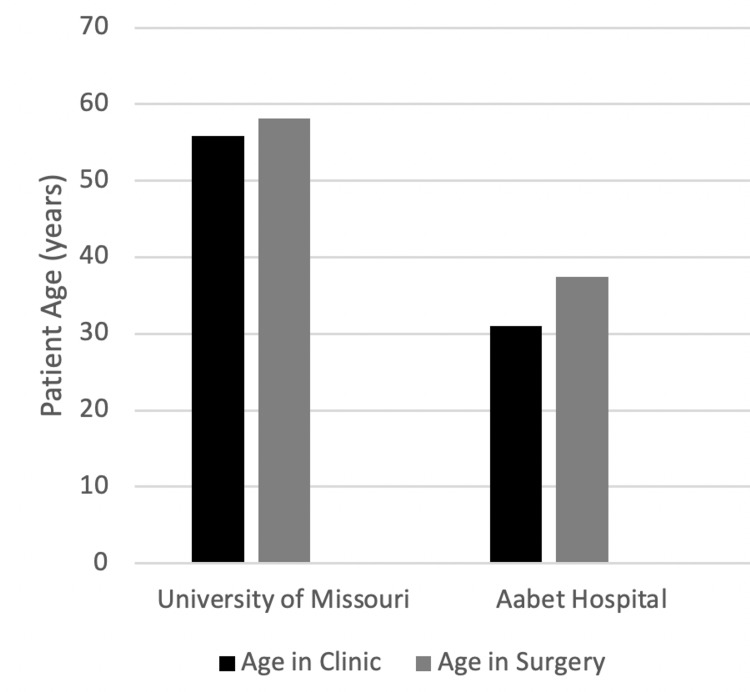
Median Patient Age

**Figure 2:**
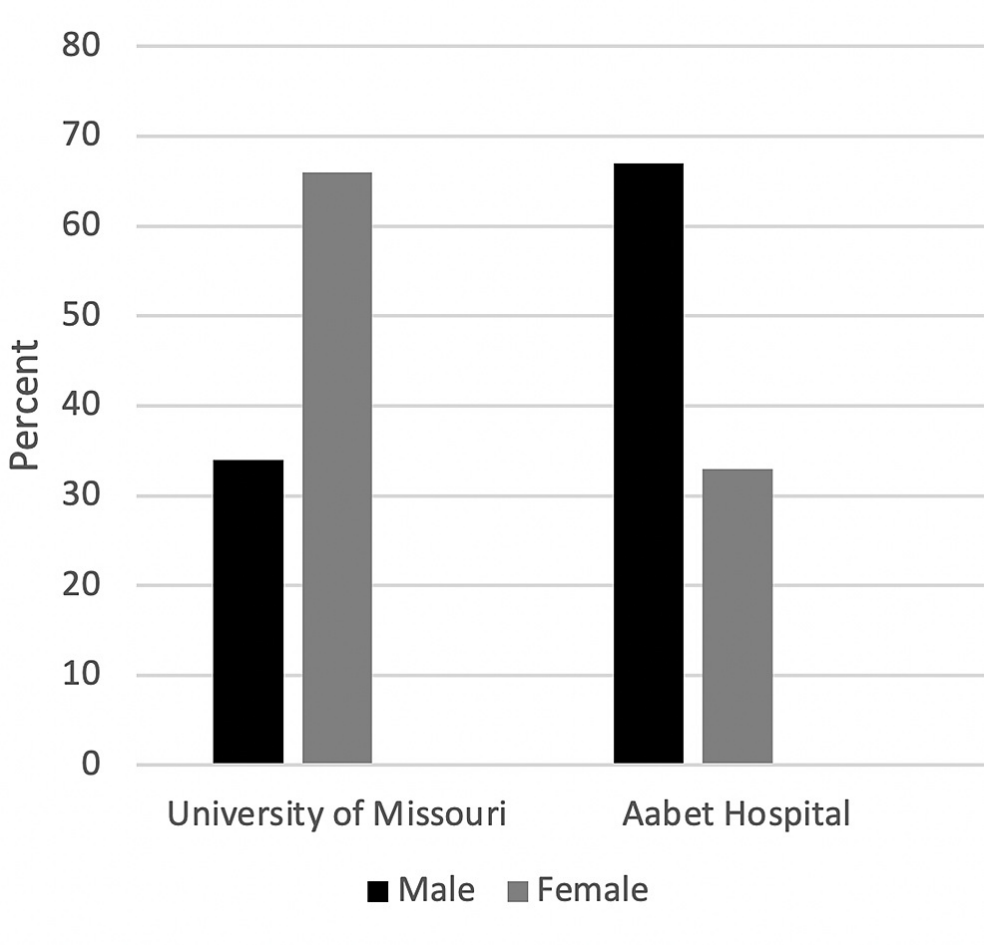
Patient Gender

The median patient age undergoing surgery was 58 years (range 21-76) at UMC and 38 years (range 1-62) in Ethiopia. A similar difference is seen in the median age of patient seen in the clinics, 56 (range 4-93) and 31 years (range 13-65), respectively (Figure 1). Patients were predominately female (66%) at UMC whereas in Ethiopia there was a male predominance (67%) (Figure 2).

**Figure 3:**
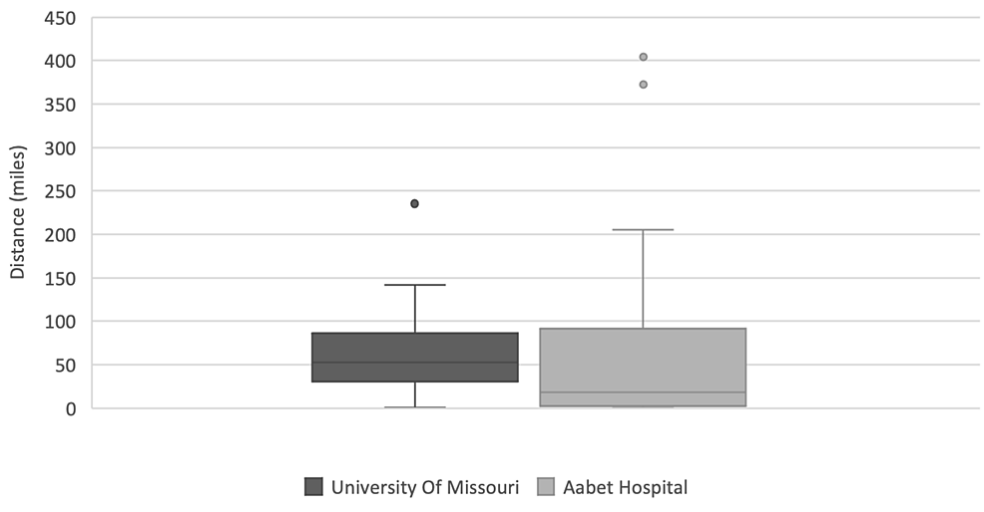
Distance Travelled by Patients

Figure 3 shows the distance travelled by patients to reach the neurosurgery clinics. At UMC the median distance was 53 miles (mean 57, range 1-235) compared to 18.6 miles (mean 85, range 1-404) at AH.

**Figure 4:**
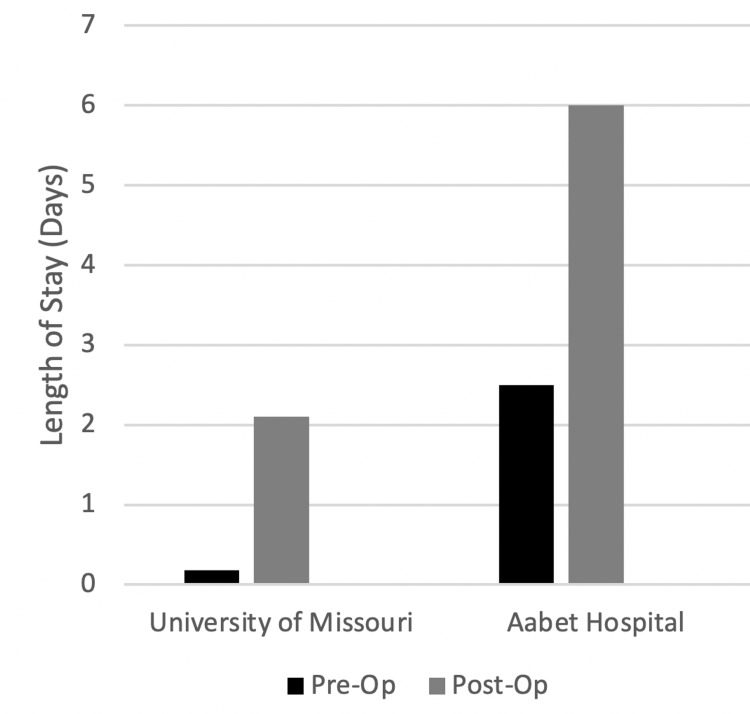
Length of Pre-Op, Post-Op Hospital Stay

**Figure 5:**
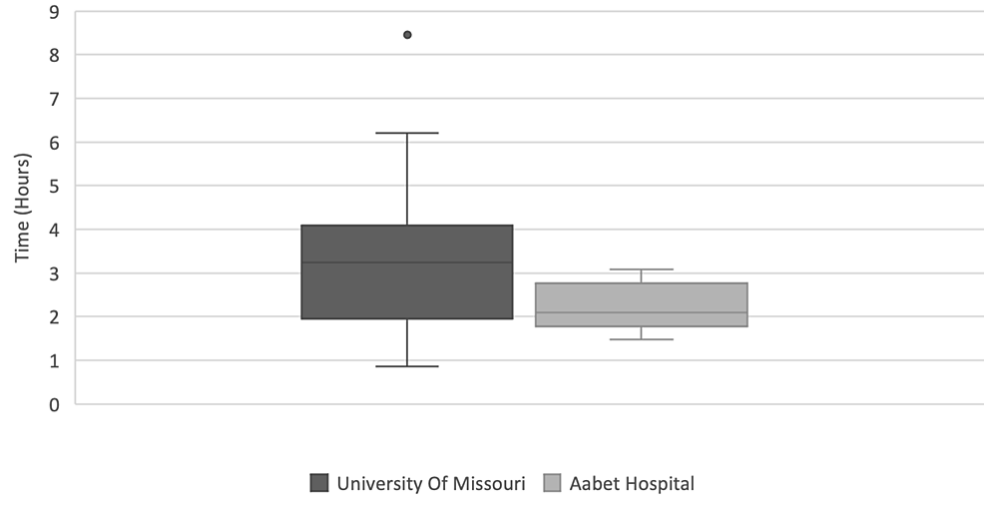
Surgical Case Length

Pre-op and post-op hospital stays were both longer at AH than UMC. The median pre-op and post-op stays at AH were 2.5 and 6 days respectively compared to 0.2 and 2.1 days at Missouri (Figure 4). No patients went home the same day as surgery at AH whereas at Missouri 7/30 (23%) went home the day of surgery. Mean hospital stays at AH were 5.1 days pre-op and 10.7 days post-op. Mean hospital stays at UMC were 3.1 days pre-op and 5.4 days post-op. As shown in Figure 5 average surgical case length was longer at UMC (median 3.2 hours, mean 3.2 hours, range 0.8-8.4) compared to AH (median 2.1 hours, mean 2.2 hours, range 1.5-3.1).

**Figure 6:**
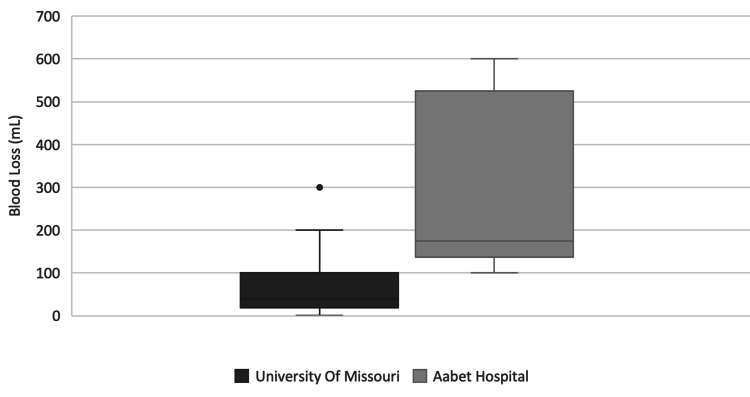
Estimated Blood Loss During Surgery

Blood loss was greater in Ethiopia with no procedures having a blood loss of less than 100 mL, and a median blood loss of 175 mL (mean 283 mL, range 100-600 mL). In comparison, the median blood loss at UMC was 50 mL (mean 74 mL, range 1-300 mL) and 17 of 30 procedures reported a blood loss of less than 100 mL (Figure 6).

## Discussion

Aabet Hospital in Addis Ababa is one of the few tertiary hospitals in Ethiopia. This hospital has one operating room for the following specialities: plastic surgery, orthopedics, neurosurgery, and trauma (which was under renovation at the time of the visit). The hospital has four attending neurosurgeons and eight residents in training. The residency is five years in duration, and virtually all residents will complete at least two years of community service as a general practitioner before starting residency.

There are 25 beds for neurosurgical patients: 20 for adults and five for pediatrics. On the weekends, residents are exclusively responsible for trauma patients and must manage them without the supervision of an attending.

The results of our follow-up study demonstrate stark differences in a multitude of elements tracked and observed. Two of these findings likely impact the individual patient and their family more than, for example, the hospital or other patients. However, two of our findings likely directly affect the individual patient as well as adding additional burdens on the hospital and, by extension, other patients.

As Table 1 shows, a clear difference among the two departments was the sheer number of patients seen at UMC compared to AH. There were 2.5 more surgeries in the week examined. Additionally, 5.5 times as many postoperative patients were seen in the clinic. The significant difference can be explained by the fact that there is only one operating room at AH designated for neurosurgical use, whereas UMC has two to three rooms. The low number of postoperative patients seen at AH is likely multifactorial but may be a direct result of the low surgical volume due to limitations in the number of operating rooms and fewer resources.

The average age of patients was quite different for both surgical patients and patients seen in follow-up. The average age of surgical patients in Columbia was 54.1, whereas it was 37.5 in Ethiopia (Figure 1). Similarly different, the average age of patients seen in the clinic was 55.9 and 31, respectively. The fact that the average patient undergoing surgery or follow-up from surgery was roughly 20 years younger in Ethiopia is concerning. It could be due to the fact that trauma, secondary to motor vehicle collision, is common in LMIC [8,9].

The findings relating to the distance a patient travels to the hospital and the amount of time a patient stayed at or near the hospital prior to surgery have significant potential to burden patients in Ethiopia far more than those seen at UMC. Patients traveled an average of 25 miles further (Figure 3) to arrive at AH, however, it is important to recognize that miles are not created equally in these two different societies. It is estimated that roughly 90% of US citizens have a car [13], and traveling is easy due to adequate infrastructure, such as highways and bridges. This is not the case in SSA [10,11]. A trip that may take less than two hours in Missouri could easily take several days to travel in Ethiopia. The reason for this is that Ethiopia has a very low rate of car ownership. In 2019, there were roughly 600,000 registered vehicles in Ethiopia, 84% of which were taxis [14]. This challenge in traveling can certainly burden a patient financially, and, as will be discussed later, even has the power to impact postoperative care.

In line with this, for patients in Ethiopia undergoing surgery at AH, the median pre-op stay was 2.5 days. During this time, at least for elective surgeries, patients await surgery near the hospital. In Columbia, this median was 0.18 days (Figure 4). In stark contrast to patients in Ethiopia, patients in Columbia who arrive for elective surgery will nearly always arrive the day of their scheduled operation. The mean pre-op stay was 5.1 and 3.1 days, respectively. The median value likely reflects a more accurate measurement for elective surgeries, whereas the mean value will reflect patients that were already at the hospital for another reason and had to undergo a neurosurgical procedure. In both cases, the average pre-op stay in Ethiopia is significantly longer when compared to Columbia. This delay poses the risk of adding a significant financial strain to the patient and their family, since the time spent waiting for the surgery is time that keeps the patient (or their family members accompanying them) from participating in the workforce. While distance traveled and preoperative stay can impact the patient, two other points of our study have clear risks of impacting both the patient and hospital.

The postoperative length of stay between the two hospitals was quite different. The average LOS at UMC was 5.4 days, whereas at AH it was 10.7 days. Again, the median value of postoperative stays is more likely to reflect elective procedures. For Ethiopia, the median was 6 days, compared to 2.1 days in Columbia (Figure 4). The reasons for the prolonged LOS at AH cannot be fully explained in this study, however, it could be due to more advanced disease in the Ethiopian population. This is supported by the prolonged wait times for surgeries to even begin. Additionally, when speaking with several of the neurosurgeons at AH, they reported that many patients refuse discharge, citing their long distance traveled, which is also supported by our findings. The surgeons report that the patients fear that if they are discharged early and a complication were to occur, they would not be able to get back to the hospital. For this reason, the surgeons keep patients admitted for longer times. Regardless of the reasons why the LOS is longer in Ethiopia, there are clear risks to both patients and the hospital that occur the longer patients are admitted.

LOS is highly reflective of proper resource utilization, since there is a disproportionate consumption of resources by patients with longer LOS [15]. Prolonged LOS has a high chance of reducing hospital bed capacity, which subsequently leads to shortages of inpatient beds, delaying elective surgery admissions, and increasing boarding in the emergency department. This can prevent timely access of treatment to critically ill patients [16,17]. While it is difficult for hospitals to influence pre-admission factors in emergency cases, identification of factors that lead to prolonged LOS in elective surgeries is vital for preparation in the management of care for such patients [16]. Recognition of factors that can lead to prolonged LOS, such as blood loss, length of surgery, transfusions, use of instrumentation, and comorbidities, can all effectuate proactive planning in the management of patients and resources [16].

Additionally, there are numerous complications to patients that can occur with prolonged LOS, such as pneumonia and infection of wounds. Such complications can increase hospital costs by over 50% [18,19]. Shortening the number of days patients spend in the hospital allows for more patients to be seen, as well as benefits the patients and their families by allowing for earlier ambulation and can lead to fewer perioperative complications [20].

Perhaps the most significant finding of our study was the difference in estimated blood loss. EBL at AH was nearly four times higher than that of UMC (283.3 mL vs 74 mL, respectively) (Figure 6). The reasons for this difference are beyond the scope of this study, however, we present several hypotheses. The significant differences in mean EBL are most likely related to resources. The use of absorbable hemostatic agents is not widely available throughout SSA as it is in the United States. Second, as the burden of trauma is higher in LMIC (as previously mentioned), this may also lead to greater blood loss. Third, patients in SSA often have a more advanced disease burden requiring more complex operations. As an example, one patient who underwent an elective operation while the two authors from UMC were there was on a rare tumor called a chordoma. The tumor spanned multiple thoracic vertebrae and abutted the descending aorta. The proximity of the tumor to the aorta required a joint operation with the cardiothoracic team. This particular operation had an EBL of 500 mL.

While it is difficult to draw conclusions about the impact this increased blood loss has on patients in Ethiopia given the low number of patients, it is certainly a topic that warrants further investigation. It is well documented that increased blood loss leads to more complications. Rajagopalan et al. found that complications of hemodynamic instability, as well as acidosis and hypothermia, were significantly increased in patients that have higher blood loss [21]. Additionally, thrombocytopenia, fever, pulmonary infections, need for tracheostomy, coagulopathy, and prolonged ICU stay (>2 days) all occurred with increasing frequency in patients with increased blood loss. This is also significant since the requirement for blood transfusion is strongly correlated with postoperative complications [22]. Furthermore, sepsis, organ failure, and death all have a positive correlation with increased transfusion [23]. Another potential problem with increased blood loss and the need for transfusion is the increased risk of transfusion-related infections in LMIC. Lack of professional oversight and insufficient regulation, minimal legislation, and ineffective implantation all pose as significant challenges to transfusion safety. The rate of transfusion-transmissible infections (TTIs) in some LMIC is already high and is probably underestimated, adding further risk to patient safety [24-28].

Because of all of this, it stands to reason that if there is four times the amount of blood loss in uncomplicated elective surgical cases in Ethiopia compared to the United States, the amount of blood loss with additional postoperative complications could be much higher in complex surgical cases.

The impact of these differences is difficult to evaluate given the limited scope of our study. However, the prolonged length of stay, both preoperatively and postoperatively, the large distances traveled, and the increased amount of blood loss all have significant potential to damage a patient, both financially and physically. The information obtained does open the possibility for further planning and management regarding neurosurgical patients in Ethiopia by providing a greater, albeit brief, overview of patient demographics and perioperative results.

One limitation of our study is the short duration we were able to collect data while at AH. Another limitation is that we were not able to directly compare the same procedures. For example, we did not compare ACDF surgery at AH versus UMC, but rather gathered information pertaining to all surgeries performed over a week for each department. While this limits the information that can be obtained from a specific operation, it does, however, provide a glimpse of what a typical week is like in each department. Strengths of the study include the fact that an author was present at AH to collect the data in real-time, which reduces the risk of errors being made in the charting process. Additionally, this study does build upon our previous work by taking a more in-depth look of a single neurosurgical department in SSA. The results of the study will allow for more specific investigation about particular points of interest (such as evaluating why EBL is higher at AH compared to UMC).

Given the results of our study, our next steps are to build upon our current work. We intend to do this by increasing the size of our data pool. We are currently in the process of developing a long-term prospective study in collaboration with the neurosurgical residents at AH which will further evaluate topics covered in this study. This will hopefully give us a greater data pool to analyze and will offer an opportunity to have robust patient information and operative data that is currently unavailable to the vast majority of physicians and surgeons in Ethiopia.

## Conclusions

Our study revealed notable differences among both neurosurgical practice and patient demographics at a single tertiary hospital in Addis Ababa, Ethiopia compared to Columbia, Missouri. This information will serve as the cornerstone for developing a system of gathering more information about neurosurgical practice and patients in Ethiopia where electronic medical records are not available. Additionally, obtaining the same information at an academic center in the United States and continuing collaboration with the neurosurgeons at Aabet Hospital will serve as a way of allowing neurosurgeons on separate continents to compare their surgeries, and patients, against another neurosurgical department.
